# Application of *w*MelPop *Wolbachia* Strain to Crash Local Populations of *Aedes aegypti*

**DOI:** 10.1371/journal.pntd.0003930

**Published:** 2015-07-23

**Authors:** Scott A. Ritchie, Michael Townsend, Chris J. Paton, Ashley G. Callahan, Ary A. Hoffmann

**Affiliations:** 1 College of Public Health, Medical and Veterinary Sciences, Australian Institute of Tropical Health and Medicine, James Cook University, Cairns, Queensland, Australia; 2 Australian Institute of Tropical Health and Medicine, James Cook University, Cairns, Queensland, Australia; 3 Pest and Disease Vector Group, Department of Genetics, Bio21 Institute, The University of Melbourne, Melbourne, Victoria, Australia; Pennsylvania State University, UNITED STATES

## Abstract

The endosymbiotic bacteria *Wolbachia pipientis* (*w*Mel strain) has been successfully established in several populations of *Aedes aegypti*, the primary dengue vector. The virulent *Wolbachia* strain *w*MelPop is known to cause several pathological impacts (increased egg mortality, life shortening, etc.) reducing overall fitness in the mosquito *Ae*. *aegypti*. Increased egg mortality could substantially reduce egg banks in areas with a lengthy monsoonal dry season, and be employed to eliminate local populations. We tested this application under semi-field cage conditions. First, we determined that *w*MelPop infection significantly reduced the survival of desiccation-resistant eggs of the dengue vector *Ae*. *aegypti*, with shade and temperature having a significant impact; nearly all *w*MelPop-infected eggs failed to hatch after 6 and 10 weeks in summer and winter conditions, respectively. In laboratory selection experiments we found that egg desiccation resistance can be increased by selection, and that this effect of *w*MelPop infection is due to the nuclear background of the host rather than *Wolbachia*. We then conducted an invasion of *w*MelPop within a semi-field cage using sustained weekly releases of *w*MelPop infected mosquitoes, with fixation achieved after 9 weeks. The egg populations *w*MelPop infected and an uninfected control were then subjected to a simulated prolonged monsoonal dry season (2.5 months) before flooding to induce hatching. The *w*MelPop infected eggs suffered significantly greater mortality than the controls, with only 0.67% and 4.35% of respective infected and uninfected eggs held in 99% shade hatching after 80 days. These studies suggest that *w*MelPop could be used to locally eliminate populations of *Ae*. *aegypti* that are exposed to prolonged dry conditions, particularly if combined with vector control.

## Author Summary

Dengue is a leading cause of morbidity in the tropics. As a commercial vaccine is not available, control or modification of the mosquito vectors is employed to prevent transmission. Strains of the endosymbiotic bacteria *Wolbachia* affect the survival and ability of the dengue vector *Aedes aegypti* to transmit dengue. The *Wolbachia* strain *w*MelPop over-replicates within *Ae*. *aegypti*, inducing strong dengue virus blocking and early mortality of both egg and adult mosquitoes. We investigated whether this life-shortening *Wolbachia* strain can be used to eliminate local populations of *Ae*. *aegypti* in a semi-field cage. Our results indicate that *Ae*. *aegypti* eggs infected with *w*MelPop died at a significantly higher rate than uninfected eggs, and were nearly eliminated during a simulated dry season of 2–3 months. This suggests that that releases of *w*MelPop could facilitate control and elimination of *Ae*. *aegypti* if used in concert with vector control.

## Introduction

Dengue is the leading cause of arboviral disease in humans. An estimated 390 million infections and 90 million clinical cases occur annually [1]. There is no commercially available vaccine, so vector control and mosquito avoidance are the only methods to limit transmission. Traditional control of the mosquito vector *Aedes aegypti* is increasingly hampered by physiological resistance against insecticides, especially synthetic pyrethroids [2–5]. Furthermore, the logistics of controlling a vector that exploits artificial containers that are often cryptic, subterranean and difficult to access (eg., elevated sites such as rain water tanks) makes effective control difficult to achieve [6–8]. The adult *Ae*. *aegypti* is endophilic, and is often found inside dark, quiet areas within premises [9]. These areas are not effectively targeted by insecticidal sprays applied by air or ground [9,10]. Collectively, vector control efforts to control dengue are generally not successful, and dengue continues to expand in breadth and reach globally.

New vector population manipulation methods have been developed to overcome the issues of traditional vector control. The release of insects infected with a dominant lethal gene (RIDL) employs the release of male *Ae*. *aegypti* that induce sterility by passing a lethal gene to wild females they mate with [11]. Serial releases of these genetically modified male mosquitoes thus lead to collapse of the resident wild population [11,12]. The vector capacity of resident populations of *Ae*. *aegypti* can also be reduced to lessen dengue transmission. Strains of the endosymbiotic bacteria *Wolbachia* were transinfected into *Ae*. *aegypti* eggs, and are then passed on maternally [13]. Male *Ae*. *aegypti* infected with *Wolbachia* induce embryo death when mating with uninfected females [13,14]. This creates a powerful drive mechanism that allows *Wolbachia* to spread naturally within a population of uninfected *Ae*. *aegypti*, and to persist once fixed [15–17]. The presence of *Wolbachia* infection also interrupts dengue (and other arboviruses) replication in *Ae*. *aegypti*, interfering with transmission [18–20].

Two virus blocking strains of *Wolbachia* (*w*Mel and *w*MelPop) have been established in *Ae*. *aegypti*. To date, populations of *w*Mel-infected *Ae*. *aegypti* have been released and established in seven localities around Cairns, Australia. The more virulent *w*MelPop over-replicates inside the mosquito, inducing a variety of physiological manifestations. These include early death (life-shortening) [13], egg mortality [21,22], reduced blood feeding [23], delayed larval development [24] and reduced overall fitness [25]. The high density of *w*MelPop probably contributes to its almost complete blocking of dengue replication and transmission in *Ae*. *aegypti* [18], but efforts to introduce this infection into wild populations have proved challenging [25], although it has been accomplished in semi-field cages [19]. One implication of high mortality, especially reduced egg longevity, is that *w*MelPop could lead to reduced population size of *Ae*. *aegypti* [26]. In areas with a pronounced monsoonal dry season, mosquitoes survive for up to several months as desiccation resistant eggs [27]. If *w*MelPop can be established in these populations during the wet season, populations might naturally die off during the dry season.

In this paper we measure the relative survival of *w*MelPop infected *Ae*. *aegypti* eggs under natural conditions. Because this strategy could be thwarted if there is evolution in *w*MelPop to counter any deleterious fitness effect, we also explore strain variation involved in egg survival. Finally, we simulate a *w*MelPop intervention from invasion to subsequent death of the eggs in a semi-field cage to test the concept that *w*MelPop could be used to crash and locally eliminate *Ae*. *aegypti* egg banks under a prolonged monsoonal dry season.

## Methods

All mosquito colonies were maintained using the standard laboratory rearing protocols [22] with minor modifications. For the egg survival experiment we used eggs from a *w*MelPop colony derived from the original microinjected *w*MelPop—CLA line which had been backcrossed to wild Cairns material for 7 generations and maintained in the laboratory with several hundred adults turned over each generation [28]. This strain was compared to uninfected material derived directly from adults or their offspring emerging from field collections of *Ae*. *aegypti* eggs. The *Ae*. *aegypti* mosquito strain used for the elimination trial and to create the desiccation selected line was a field-sourced population of the *w*MelPop *Wolbachia* infected line (AOMB) [24]. This colony was initiated from 18 infected females collected from Machans Beach 0.6 months after releases of *w*MelPop had been terminated there. The infection in this colony had therefore been exposed to field conditions during breeding cycles. It was subsequently maintained in the laboratory at >200 adults. Experiments with this colony [24] indicated that the deleterious host fitness effects that *w*MelPop is known to have on longevity, fecundity and egg viability were also detectable in this colony. Prior to the start of experiments, AOMB was backcrossed for three generations with adults emerging from wildtype eggs from Cairns, QLD.

### Egg survival

**Fig 1:**
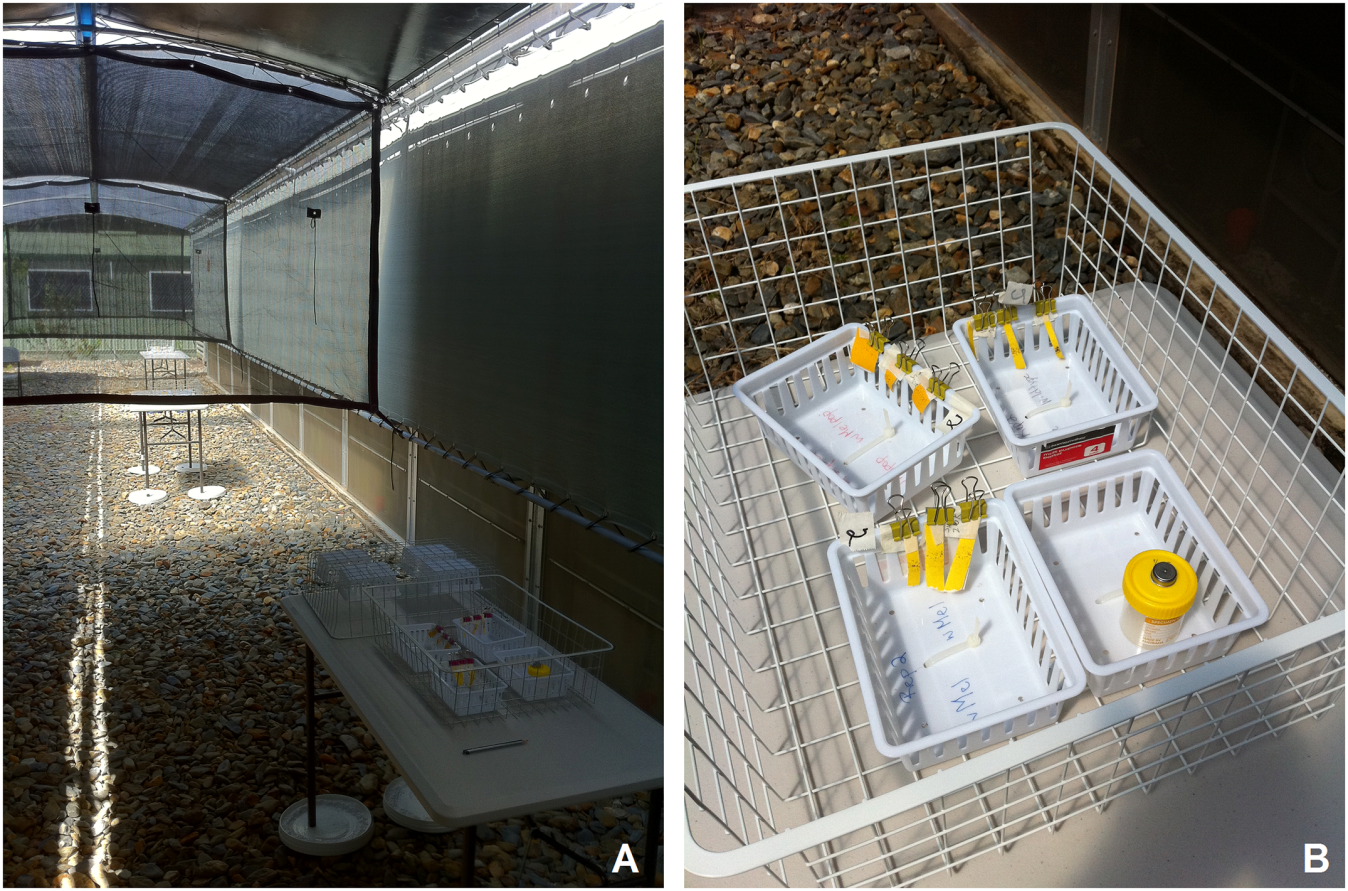
A. Shade arena for egg exposure trials showing 99%, 70% and 30% shade sections, and B. egg strips with data logger.

A protective awning with three different shade regimes was built to house mosquito eggs in the survival study. The awning (17 m x 3.3 m) was built between 2 semi-field cages [29] and divided into three sections that provided 30%, 70% and 99% shade (Fig 1). The awning roof consisted of waterproof translucent vinyl to protect eggs from rainfall. Two 5.6 m sections of the vinyl had an interior piece of 40% and 95% shade cloth to provide additional shade to create the 70% and 99% shade; the section with vinyl alone provided 30% shade. Temperatures and relative humidity within each section were monitored using data loggers (Hygrochron iButton DS1923, Dallas Semiconductors) set atop and below the table, supplemented with data from the Bureau of Meteorology (BOM) site 15 km SE of the site when loggers failed. Preliminary trials indicated that ambient temperatures (under table) in the 70% and 99% shade (S1 Fig) were nearly identical to screen height temperatures at the Cairns Airport BOM site. However, loggers on the table that were exposed to sunlight (30% and 70% shade) experienced a spike in temperatures that exceeded 50°C and 60°C in the winter and summer, respectively (S2 Fig).

Egg strips were obtained by exposing sandpaper strips to gravid mosquitoes. Eggs were allowed to incubate within the flooded oviposition cup for 3 days, then dried. Viable (non flattened) eggs on strips were counted and each strip labeled with egg count using masking tape on a bulldog clip). Eggs strips were attached vertically to the inside of a wire mesh basket that was set on a card table within each section (Fig 1). The legs of the table were set inside a saucer containing talcum powder to prevent ants from invading the table and predating eggs. Three egg strips were randomly picked and hatched in a dilute yeast solution at 0, 1, 2, 3, 4, 6, 8, 12 and 16 weeks post exposure. The experiment was conducted in the cool dry (10 May– 30 Aug. 2012) and warm dry season (11 Oct. 2012–3 Jan. 2013). Percent hatch was calculated as the number of hatched eggs (1^st^ instar larvae) divided by total viable eggs.

To assess changes in hatch rates across time in the summer and winter egg quiescence experiment, we considered rates observed in the first 6 weeks or less and then ran General Linear Models (GLMs) examining the impact of strain and shade on hatch rates, with time treated as a linear and quadratic variable. We were particularly interested in the interaction between time and strain reflecting changes in hatch rate over time. GLMs were run with and without angularly transforming hatch rates but only the latter are represented because the conclusions were identical.

### Elimination trial

We conducted a trial to see if establishing a fixed population of *w*MelPop infected *Ae*. *aegypti* could be used to crash the viable egg bank during the dry season and potentially eliminate the local population under semi-field cage conditions. Two semi-field cages (4 x 7 x 4 m) were populated with wild (F1-2) Cairns *Ae*. *aegypti*. Each cage contained 4 10-L buckets and 4 potplants with flooded potplant base (PPB). Three times per week a volunteer sat in the cage for 10 min to bloodfed mosquitoes; 1 pellet of lucerne was added to each bucket and the potplant base was refilled with water. Buckets and PPBs were flooded weekly to hatch new eggs. The populations were introduced into the cage on 28 April 2013 and allowed to establish for 4 weeks. Then populations were crashed (simulated source reduction campaign) by emptying 3 of the 4 buckets and PPBs to kill immatures then spraying the inside of each with 4% sodium hypochlorite (commercial bleach) to kill eggs [30]. One week after population suppression, PPB and buckets were refilled with water. Mean pupal counts were used to estimate the adult population in both cages by integrating estimated adult populations for 0.8 and 0.9 daily survival over 14 days [31,32]. Single Biogents Sentinel traps (BGS) were run for 3 hr in each cage 2–3 times/week to monitor adult populations. We used the relative change in BGS captures during the 2 weeks before and after releases of *w*MelPop-infected mosquitoes to estimate the size of the released cohort [33].

On 6 June 2013 weekly release of *w*MelPop infected mosquitoes began in the treatment cage. A colony of AOMB *w*MelPop cultured at a constant temperature was the source of release material. Two cups of 50 (1:1 male:female) adult *Ae*. *aegypti* infected with *w*MelPop were released into the north (treatment cage). This equated to a relative release ratio of 1.4 and 2.0 for female and male infected mosquitoes, respectively, based on the relative change in BGS captures from 2 weeks before and after release [33]. Mosquito populations were also monitored in both cages by counting pupae in buckets and PPBs once week. Mosquitoes in the control and treatment cage were monitored for *Wolbachia* infection by PCR analysis of sample of up to 30 larvae/week; larvae were reared to adults for PCR. We also conducted another source reduction (3/4 of buckets and PPBs treated as before) supplemented with removal of adults using a sweepnet on 5 July. Releases of *Wolbachia* infected mosquitoes continued until 100% of sampled mosquitoes in the treatment cage were infected with *w*MelPop for two successive weeks; the last release was on 26 Sept. 2013.

We characterized the infection frequency in the cage by testing samples of 30–90 adults (collected as larvae) for the *Wolbachia* infection. DNA was extracted from adult mosquitoes by using a Chelex 100 resin (Bio-Rad Laboratories, Hercules, CA) extraction method. 38 mosquitoes were ground in 3 L of proteinase K (20 mg/mL) (Roche Diagnostics Australia Pty. Ltd., Castle Hill New South Wales, Australia) and 250 L of 5% Chelex solution, incubated at 65°C for 1 hour, followed by incubation for 10 minutes at 90°C and storage at -20°C. *Wolbachia* infection status was determined by using methods developed for a Roche LightCycler 480 [34] with a modification to the primers used as described elsewhere [25]. Quantitative real-time polymerase chain reaction (PCR) was used to amplify three markers with three primer sets: a mosquito primer set to detect mosquitoes from the *Aedes* genus, *Ae*. *aegypti* specific primers to differentiate *Ae*. *aegypti* from other *Aedes* species, and *Wolbachia* specific primers to determine *Wolbachia* infection status, as well as density. PCR conditions were as follows: 95°C for 10 minutes, 40 cycles of 95°C for 5 seconds, 58°C for 15 seconds and 72°C for 15 seconds, ending with a 95°C 1-minute heating followed by cool down to 40°C for 20 seconds before raising to 65°C. A melting curve analysis was performed via a gradual increase of temperature from 65°C to 95°C. Diagnosis was based on crossing-point values for the PCR and melting temperature from high resolution melt analysis.

We tested the potential for *w*MelPop to eliminate the field cage population of *Ae*. *aegypti*. Mosquitoes in both the treatment and control cages were allowed oviposit for three weeks after the last release. To provide a substrate that could be easily divided into separate egg cohorts to measure survival, we placed a roughened plastic liner inside each bucket for oviposition [30]. Sections of each liner were then cut off with scissors, viable eggs counted microscopically, and placed within the 70% and 99% shade sections of the awning used to measure egg survival. Strips of *w*MelPop infected and uninfected eggs were hatched in dilute yeast solution after 29, 59 and 80 days exposure (7 Nov, 16 Dec and 6 Jan). Strips were dried for 1 week then reflooded to hatch any viable eggs not initially hatched. Percent hatch was calculated as with the egg survival study. In the final hatch all potplant bases and buckets were flooded to see if any eggs that may have been laid on the container rather than the ovistrip survived. We compared the total percent hatch between treatment and control for exposure period using chi square.

### Selection and backcrossing

Selection experiments were conducted to examine if egg desiccation resistance was due to a nuclear or *Wolbachia* effect. Eggs were collected daily for four days using sandpaper ovistrips and were fully conditioned as per the standard protocol [22]. All ovistrips were placed into a desiccation chamber set at ~60% Relative Humidity (RH) using a saturated solution of sodium bromide [22]. RH was monitored using a hygrochron (1-wire, iButton.com).

To select a *w*MelPop desiccation resistant line (AOMB-DS), we were aiming to obtain around 10% hatch rate over four rounds of selection. Previous experiments have shown that a 10% hatch rate occurred around Day 20–21. Therefore, the ovistrips were divided into batches and hatched over a range of days (18–26) to obtain a hatch rate close to 10%. Eggs were hatched using Reverse Osmosis (RO) water, Tetramin fish food and yeast [22]. Hatch rate was determined by (number of eggs/number of larvae) x 100. In each generation of selection, at least 100 eggs were used in the next generation. Each round of selection was followed by a non-selected generation to increase the number of adults for producing eggs available for selection in the next generation. There were four rounds of selection and the colony was then maintained in the laboratory over several generations.

**Fig 2:**
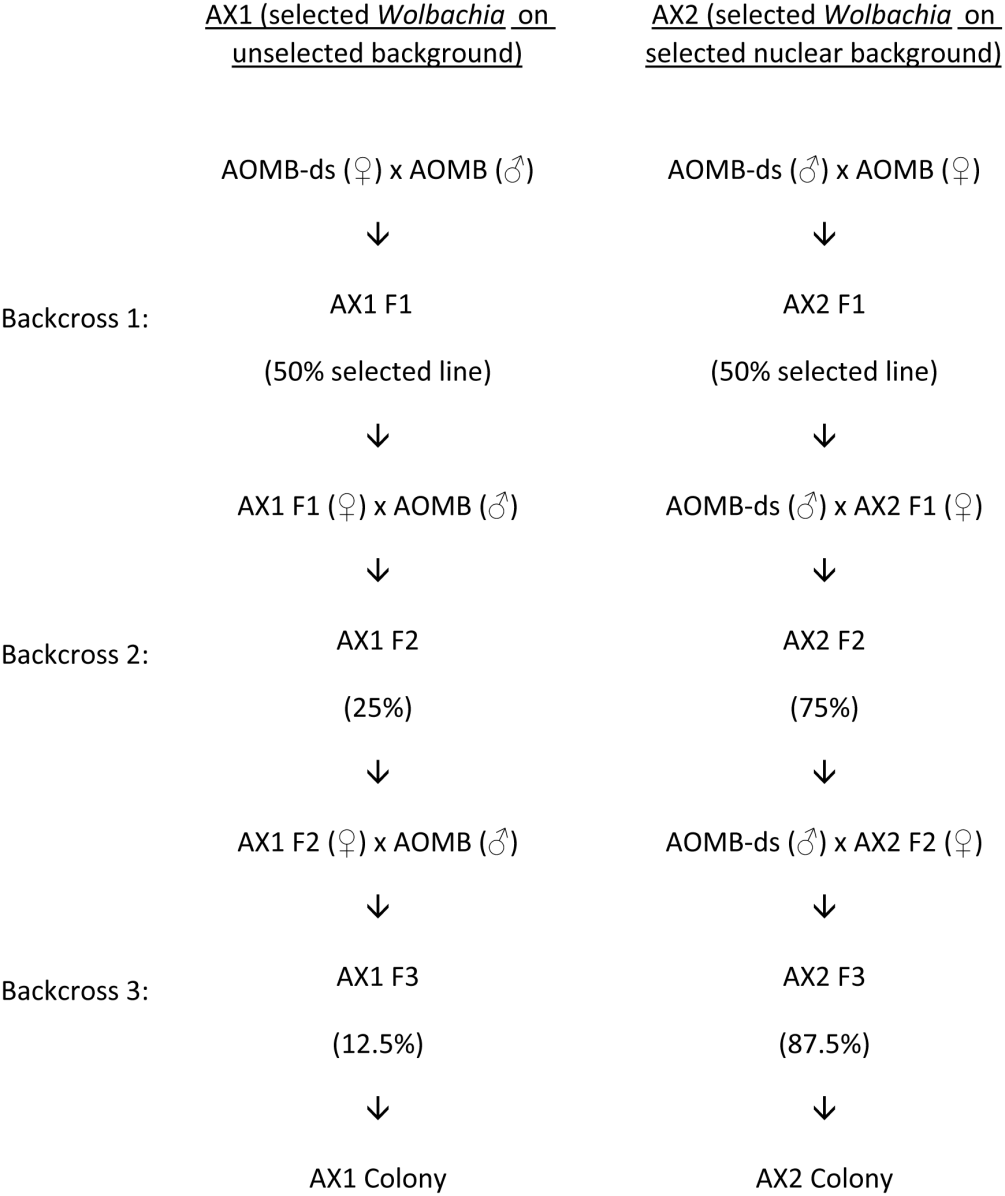
Crossing scheme to produce colonies with the selected *Wolbachia* background (AX1) or selected nuclear background (AX2).

To determine if the selection response in AOMB-DS was due to a nuclear or *Wolbachia* effect, the selected line was crossed to unselected lines. Two new lines (AX1, AX2) were created by backcrossing AOMB-DS to males or females of the original AOMB line. Backcrossing was continued for three generations to place *Wolbachia* from the selected line onto the nuclear background of the infected (AX1) stock (Fig 2) or to place *Wolbachia* from the unselected line onto the nuclear background of the selected infected line (AX2).

To test for viability, eggs were collected over four day periods from the AOMB-DS, AOMB, and C20 colonies as well as the backcrossed AX1 and AX2 lines. These were placed in the 60% desiccation chamber once conditioned. Three days later, eggs were cut into 10 batches of at least 25 eggs then counted and hatched into separate cups filled with 170 ml RO water and TetraMin fish food. Larvae were counted one week later as an estimate of viability. This process was carried out on eggs aged for 3 days and then repeated after 10, 17, 24, 31, 38, 45, 52, 59 and 66 days [22].

Following selection, to compare the association between hatch rates and time in the different strains, we truncated the changes in hatch rates at 32 days (ie before all the unselected *w*MelPop eggs had become non-viable) and ran GLMs that included linear and quadratic terms for time as well as line type. These parameters provided an adequate fit to the data or each line (R^2^>0.90). We then considered whether lines differed for estimated parameters in these models. Because multiple strains were compared we computed parameter estimates for the different treatments and interactions and tested their significance using bootstrapped confidence intervals estimated in IBM SPSS Statistics 22.

To determine if the selection response in AOMB-DS was due to a nuclear or *Wolbachia* effect, the selected line was crossed to unselected lines. Two new lines (AX1, AX2) were created by backcrossing AOMB-DS to males or females of the original AOMB line. Backcrossing was continued for three generations to place *Wolbachia* from the selected line onto the nuclear background of the infected (AX1) stock (Fig 2) or to place *Wolbachia* from the unselected line onto the nuclear background of the selected infected line (AX2).

To test for viability, eggs were collected over four day periods from the AOMB-DS, AOMB, and C20 colonies as well as the backcrossed AX1 and AX2 lines. These were placed in the 60% desiccation chamber once conditioned. Three days later, eggs were cut into 10 batches of at least 25 eggs then counted and hatched into separate cups filled with 170ml RO water and TetraMin fish food. Larvae were counted one week later as an estimate of viability. This process was carried out on eggs aged for 3 days and then repeated after 10, 17, 24, 31, 38, 45, 52, 59 and 66 days [22].

### Data repository

All summary data can be accessed in Dryad Digital Repository at http://dx.doi.org/10.5061/dryad.3vg33 [35].

## Results

### Egg survival

**Fig 3:**
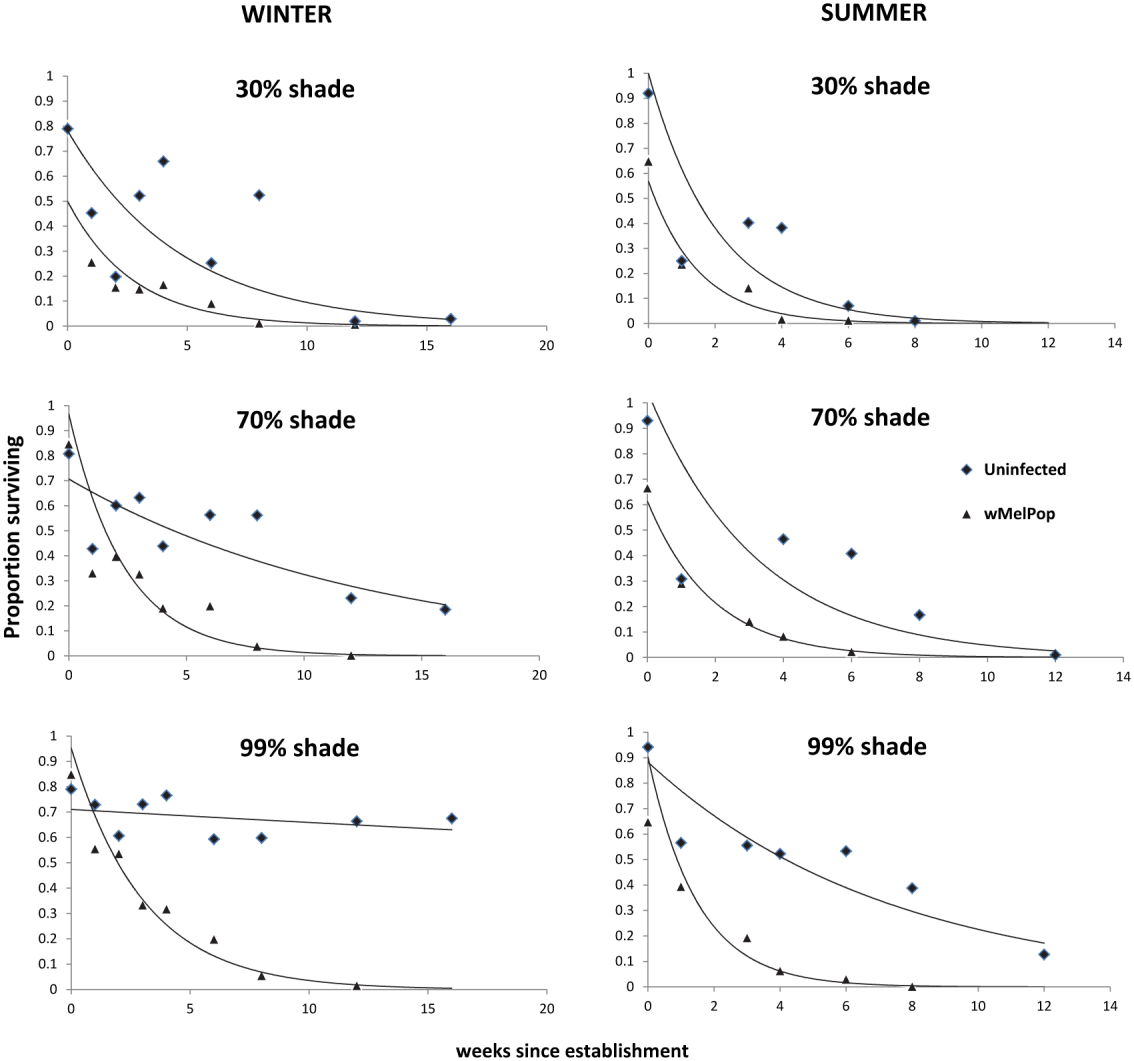
Hatch rate (survival) of *Ae*. *aegypti* eggs infected with *w*MelPop and an uninfected control held in 30%, 70% and 99% shade in winter and summer.

The *w*MelPop infected *Ae*. *aegypti* eggs exposed to a range of shade (Fig 1) suffered higher mortality rates than uninfected eggs (Fig 3). Mortality (decrease in % hatch) was highest in summer and in sun exposed (30%, 70% shade) eggs. Indeed, mortality of *w*MelPop infected eggs was nearly 100% after 8 weeks in all shade groups in summer. Uninfected eggs also suffered high mortality, but only in the highly exposed 30% shade treatment. The GLM on data from the summer experiment indicated a significant effect of shade and strain overall as well as time as a linear and quadratic term (Table 1). Interactions between strain and the time variables were not significant, although the quadratic—strain interaction approached significant. Increased shade led to lower viability across time (Fig 3) and the parameter estimates indicated significant differences between the 99% shade and the 30% treatment, but there was no interaction between shade and strain or other interactions with shade (all P>0.25). The *w*MelPop strain had lower viability overall and tended to show a more rapid decrease in viability over time.

**Table 1:** Effect of shade and strain on hatch rates (angular transformed) of eggs across weeks (evaluated as linear and quadratic components).

Source	df	Mean Square	F	*P*
Summer				
Shade	2	0.485	8.526	<0.001
Strain	1	0.876	15.409	<0.001
Week (linear)	1	5.766	101.397	<0.001
Week (quadratic)	1	2.434	42.799	<0.001
Strain by Week	1	0.053	0.924	0.338
Strain by Week (quadratic)	1	0.174	3.063	0.082
Error	172	0.057		
Winter				
Shade	2	0.985	19.865	<0.001
Strain	1	0.007	0.144	0.705
Week (linear)	1	2.184	44.058	<0.001
Week (quadratic)	1	0.531	10.707	0.001
Strain by Week	1	0.861	17.364	<0.001
Strain by Week (quadratic)	1	0.515	10.385	0.002
Error	154	0.050		

For the winter experiment, the strain-shade interaction effects were also non-significant (P > 0.25). All other effects except for the main effect of strain were highly significant (Table 1). There were different patterns of changes in the hatch rates of strains across weeks, with linear and quadratic components. The *w*MelPop infection had a sharper drop in hatch rates compared to the uninfected line (Fig 3). The temperature in the winter trial ranged from ca. 15–28°C, with slightly higher temperatures in the 30% shade section (S1 Fig). In winter, temperatures recorded from loggers on tables exposed to direct sunlight (30% and 70% shade) peaked at 40–50°C in the afternoon (S2 Fig). Relative humidity was generally high, ranging from ca. 50% to nearly 100% at night, although the maximum values are likely too high (S3 Fig). In summer, temperature range increased to ca. 20–35°C (S4 Fig), with exposed loggers peaking at 40–60°C at mid day for 30% and 70% shade (S2 Fig). Relative humidity ranged from 50% to 90% (S3 Fig).

### Elimination trial

**Fig 4:**
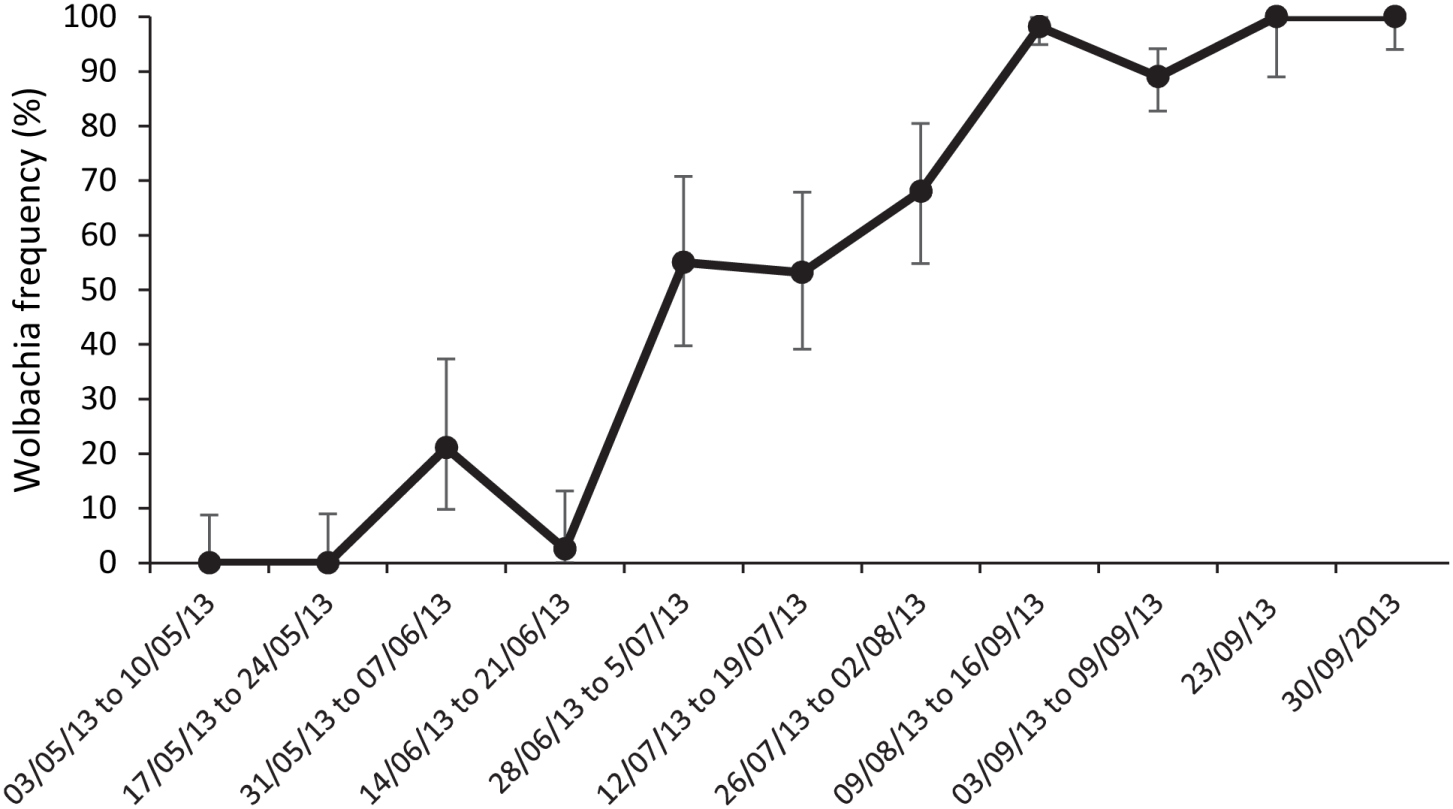
Frequency of infection in the semi-field cage with error bars representing 95% confidence intervals. Weekly releases of 50 male/female *w*MelPop infected mosquitoes began on 6 June and continued through 23 Sept. Vector control consisting of treatment of 75% of breeding containers was conducted on 30 May and 5 July. Adult mosquitoes (> 40 sample) reared from larvae/pupae were assayed for *w*MelPop infection by PCR.

Releases resulted in the *w*MelPop infection gradually moving to fixation within the semi-field cage. Two successive weeks of 100% *w*MelPop infection rates were achieved after 16 consecutive weekly releases and two source reduction/vector control interventions (Fig 4).

The mean temperature in the 70% and 99% shade sections was 26.2°C and 25.4°C, respectively, and ranged from 20–35°C (S5 Fig). Relative humidity ranged from 50% to 95%. While we did not record temperatures in the exposed data loggers (set on tables), results from the earlier egg survival study indicate that temperatures of 50°C would have occurred in the 70% shade section for a few hours on sunny days (S2 Fig). Mean (± SE) pupal counts for individual PPB and buckets in the *w*MelPop and uninfected cages were 0.62 ± 0.96 and 1.06 ± 1.37; and 28.9 ± 30.6 and 50.4 ± 27.2, respectively. Collectively, the mean pupal production/day was 59 ± 49 and 103 ± 46 for the *w*MelPop and control cages, respectively. Based on a 0.8 and 0.9 adult daily survival (DS), this would equate to a mean adult population, using the integration method [30], of 280–451 and 490–790 adults for the *w*MelPop and uninfected cages, respectively.

**Fig 5:**
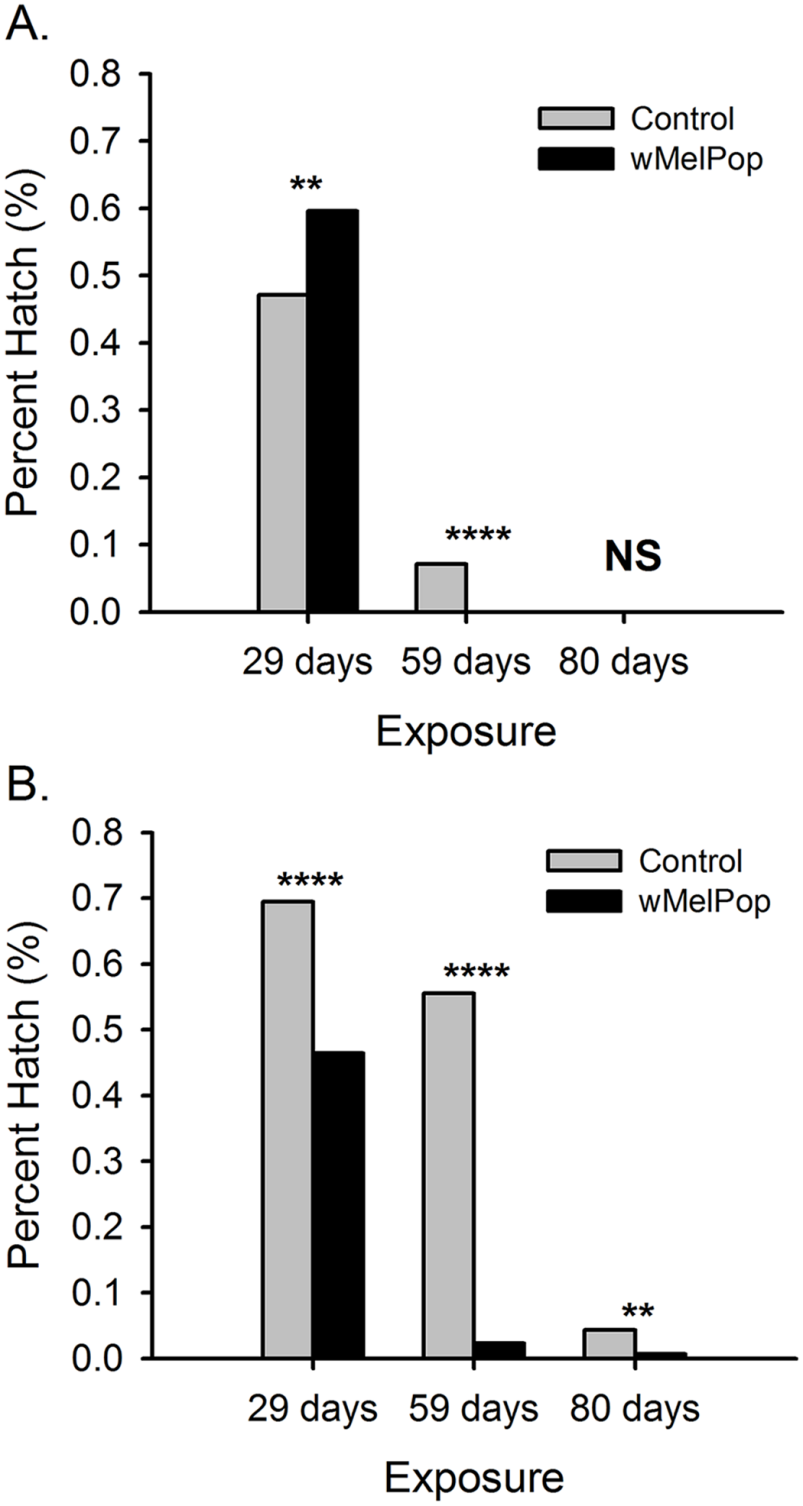
Egg survival (egg hatch) of wild and *w*MelPop infected *Ae*. *aegypti* eggs held outdoors in A. 70% and B. 99% shade from 18 Oct. 2013–6 Jan. 2014. ** and **** denote P < 0.01 and P < 0.0001, respectively.

Eggs infected with *w*MelPop suffered significantly higher mortality than uninfected eggs for each exposure period except for 80 days at 70% shade, where no eggs hatched for either cohort (Fig 5). *w*MelPop infected egg survival dropped noticeably after 59 days, and only 3/443 (0.67%) eggs hatched from the 99% shade cohort after 80 days. Mortality was also high in the uninfected eggs, but 7/154 (4.4%) eggs hatched in the 99% shade cohort. No *w*MelPop infected eggs hatched from the PPB and buckets flooded at the end of the trial, while 9 hatched from a PPB with uninfected eggs.

### Selection and backcrossing

After four rounds of selection for desiccation resistant mosquitoes, there was an increase in the time required to produce a hatch rate of around 10% from around 17 days to almost 40 days (Table 2). This followed a rapid extension after two rounds of selection.

**Table 2:** Response to selection for desiccation resistance through four rounds of selection.

Round of selection	Hatch rates	Number of days to reach rates
1	2.01–17.2%	17–24
2	2.19–10.2%	21–24
3	1.8–10.7%	37–40
4	1.8–10.7%	36–39

**Fig 6:**
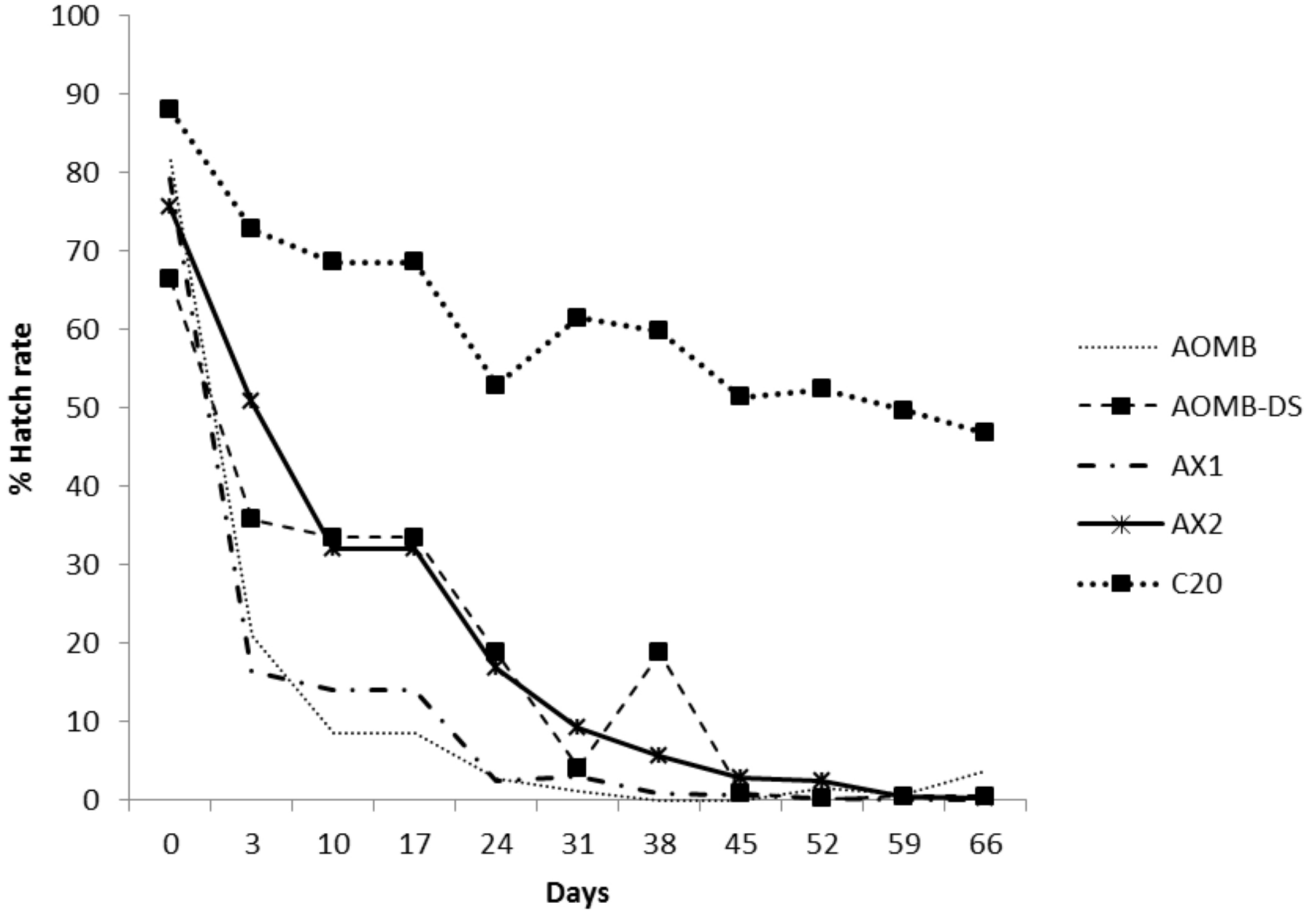
Changes in the hatch rates of the lines when eggs are maintained in a quiescent stage for different lengths of time before being hatched. An uninfected line (C20; top line) was included as a control. The other lines are the infected *w*MelPop line (AOMB), the same line after four rounds of selection (AOMB-DS), and backcross lines to place the *w*MelPop infection from the selected line onto the nuclear background of the unselected line (AX1) or the infection from the unselected line onto the nuclear background of the selected line (AX2).

In the backcrossed lines, the AOMB-DS line maintained a higher hatch rate in aged eggs compared to the AOMB line despite several generations without selection (Fig 6). The AX2 line showed a similar pattern to that of the AOMB-DS line. As expected, the uninfected C20 had the highest hatch rate. The unselected AOMB and AX1 lines had similar low hatch rates. The GLM showed that there was a significant effect of line on hatch rate (F_(4, 285)_ = 4.097, P = 0.003) and there were also significant interactions between line and the linear time component (F_(4,285)_ = 6.974, P<0.001) and between line and the quadratic time component (F_(4,285)_ = 5.042, P = 0.001). These analyses indicate differences among strains in hatch rate changes across time involving both a linear and non-linear component. The time effects point to an increase in the hatch rate (survival) of the selected line with the *w*MelPop infection associated with the nuclear background of the line rather than the *Wolbachia* background. The AX1 line and AOMB did not differ in any way and all parameter estimates overlapped for these strains (main effects of strain, interactions with linear and quadratic time components). This was also the case for AX2 and AOMB-DS. In contrast, the linear parameter for AX2 and week (-0.103) fell outside the 95% confidence intervals for the equivalent value for AOMB (-0.582, -0.192) and this was also the case for the quadratic component (0.001) which fell outside the 95% confidence intervals for AOMB (0.015, 0.101). The quadratic component also differed between the selected and unselected AOMB lines (AOMB, 0.058, 95% CIs 0.015, 0.101; AOMB-DS, -0.031, 95% -0.074, 0.013) as did the linear component (AOMB, -0.387, 95% CIs -0.582, -0.192; AOMB-DS, 0.051, 95% -0.144, 0.246).

## Discussion

*Aedes aegypti* eggs infected with *w*MelPop suffer significantly higher mortality than uninfected eggs. Nearly all of the infected eggs failed to hatch after 2 months for all shade and temperature exposures (Fig 2). Our semi-field cage experiment indicted that if releases of *w*MelPop successfully establish fixation, the population will potentially collapse during prolonged dry periods. Indeed, the hatching rate (survival) of *w*MelPop infected eggs was significantly lower after 59 and 80 days exposure (Fig 3). This points to the feasibility of using releases of *w*MelPop infected *Ae*. *aegypti* to eliminate local populations of *Ae*. *aegypti* and confirms the potential of this approach as first proposed and modelled in Rasic et al. [26].

Nevertheless, using *w*MelPop releases for elimination of *Ae*. *aegypti* populations will be more difficult than invasions involving *w*Mel releases. To obtain fixation in the semi-field cage, we conducted 16 weeks of releases and two rounds of vector control. This is considerably more difficult than the invasions of *w*Mel that have successfully established in Cairns after 11 weeks of releases, and at a faster rate in semi-field cages [17,19]. Indeed, sustained releases of *w*MelPop-infected *Ae*. *aegypti* failed to establish fixation by *w*MelPop at two field sites in Cairns in 2012 [25]. We could have improved efficacy of the releases by increasing the number of infected mosquitoes released. At the start of releases, the population in the treatment cage, based on pupal integration, was ca. 200 adults. We released 100 per week, or a release rate equivalent to 50% of uninfected adult population, although BGS captures suggested the initial 2 weeks of release cohorts were 150% of the uninfected population. The successful *w*Mel invasions in Cairns involved releases at numbers roughly equivalent to wild populations. Higher releases rates should have improved the speed at which *w*MelPop became fixed in our semi-field cage.

The use of *w*MelPop to eliminate local populations of *Ae*. *aegypti* will likely be limited by several constraints. It took 16 weeks of releases, with 2 cycles of vector control, to fix *w*MelPop in a small semi-field cage. Thus, releases without vector control, population suppression or another mechanism to assist invasion like the use of insecticide resistance are likely to be challenging [36]. The site will likely need to be isolated to avoid rapid re-infestation from adjacent areas, and will likely need to be relatively small, such as an isolated township [26]. Also the locale must have a prolonged dry period to facilitate egg death, as modelled in Rasic et al. [26]. Indeed, there were 4 viable infected eggs (3 from a bucket, and 1 from a PPB) after 80 days in the full shade cohort, suggesting that *w*MelPop may persist at a very low level requiring additional vector control for elimination. Finally, candidate areas need to be focused on the elimination of *Ae*. *aegypti*, rather than simply a reduction of dengue risk, which will be more easily achieved by releases of *w*Mel rather than *w*MelPop. The data generated from this study could be used to identify geographic areas through modelling where the use of virulent *Wolbachia* and vector control for population suppression would be most effective.

Vector control and population manipulation can be used to facilitate *w*MelPop releases [36]. Improved vector control methods that target cryptic breeding sites, such as the use of auto-dissemination application of pyriproxyfen [37], could temporarily remove cryptic foci of uninfected mosquitoes, enhancing invasion. Other temporary vector control methods, such as use of bleach to treat larval habitat [30] and non-persistent adulticides such as metofluthrin [38], could provide a quick knockdown and avoid residual effects that would impact released mosquitoes (crash and release strategy). A novel approach would be to couple genes for pesticide resistance to *w*MelPop, in effect engendering protection of the *w*MelPop mosquitoes from insecticides [39], although this approach may be coupled with the fear of introducing resistant strains, and could be difficult to sell to the public, regulators and government officials.

While the *w*MelPop infection has deleterious effects on egg viability when these are in a dried state, these effects are attenuated by strong selection. The crosses point to this attenuation being due to a nuclear background effect (Fig 6). Previous research has shown that the expression of *Wolbachia* effects on the host can be influenced by host background. Perhaps the most dramatic example of this is where *Wolbachia* causing cytoplasmic incompatibility or male killing in one host and then transfected into a different host species no longer expresses the same phenotype [40]. In addition, lifespan effects generated by *Wolbachia* in *Drosophila melanogaste*r are altered through longevity selection and involve the nuclear background [41]. Once *w*MelPop have been successfully introduced into populations, evolution in *Wolbachia* may attenuate fitness effects, as recently documented in Drosophila [42] although this is clearly not always the case. Evolution seems likely to change the nuclear background and lead to attenuation, but this process may be countered by other selective pressures.

Does use of a virulent *Wolbachia* strain such as *w*MelPop to eliminate local *Ae*. *aegypti* populations have advantages over traditional releases of sterile (sterile insect technique (SIT)) or incompatible (incompatible insect technique (IIT)) males? Certainly use of *Wolbachia* eliminates the need for costly irradiation equipment and any potential fitness cost associated with irradiating males. Reared *Wolbachia*-infected cohorts do not need to be sexed before release, and the relative numbers of released insects should be much lower that SIT programs where overwhelming release ratios of high quality males are required [43]. There is no sudden population “bounce back” if releases of *Wolbachia* infected mosquitoes stopped (assuming *Wolbachia* is nearly fixed in the population) unlike SIT/IIT where residual wild populations can rapidly rebound. *Wolbachia* is seen as a biological control program with generally strong community support [44], whereas some IIT programs involve genetically modified organisms, and may be relatively difficult to obtain public and regulatory support for. Disadvantages of use of virulent *Wolbachia* include the release of biting females, of the need to create a suitable infected line, and the potential requirement to conduct complimentary vector control.

The survival of uninfected *Ae*. *aegypti* eggs exposed to direct sun (30 and 70% shade) deserves comment. While egg survival was highest in full shade, many eggs exposed to sunlight (30% and 70% shade) survived several weeks (Fig 2). *Ae*. *aegypti* eggs exposed to temperatures > 46°C suffer rapid mortality [45,46]. Our data loggers recorded mid day temperature spikes of up to 40°C to 60°C, 20–30°C higher than ambient temperature, lasting several hours (S2 Fig). We suspect that the loggers, oriented horizontally on top of the sample jar (Fig 1), were subject to high solar gain and heating, and thus misleadingly high temperature readings. The *Aedes* eggs, on the other hand, are oriented vertically, minimizing their silhouette area when the sun is overhead and thus reducing solar gain. The small size of eggs would also facilitate cooling by the air.

In summary, we have established the effects of the *w*MelPop infection on egg mortality when these are maintained in a dried state under semi-field conditions. This provides the possibility of using *w*MelPop to suppress and even eliminate mosquito populations in relatively isolated populations particularly where there is a long dry season.

## Supporting Information

S1 Fig Temperature (daily high, low and average from logger in shade) in 30%, 70% and 99% shade sections of the egg exposure unit in winter. (TIFF)

S2 Fig Temperature from data loggers exposed to sunlight in egg exposure unit in winter and summer. (TIFF)

S3 Fig Relative humidity in 99% shade section of the egg exposure unit during the egg survival experiment. (TIFF)

S4 Fig Temperature (daily high, low and average from logger in shade) in 30%, 70% and 99% shade sections of the egg exposure unit in summer. (TIFF)

S5 Fig Temperature and relative humidity (99% shade) in 70% and 99% section of egg exposure unit during the *w*MelPop elimination trial. (TIFF)
