# Supplementary figures and images for: Application of wMelPop Wolbachia Strain to Crash Local Populations of Aedes aegypti
Source: PLoS Negl Trop Dis. 2015 Jul 23;9(7):e0003930. doi: 10.1371/journal.pntd.0003930 (PMC4512704; doi:10.1371/journal.pntd.0003930)

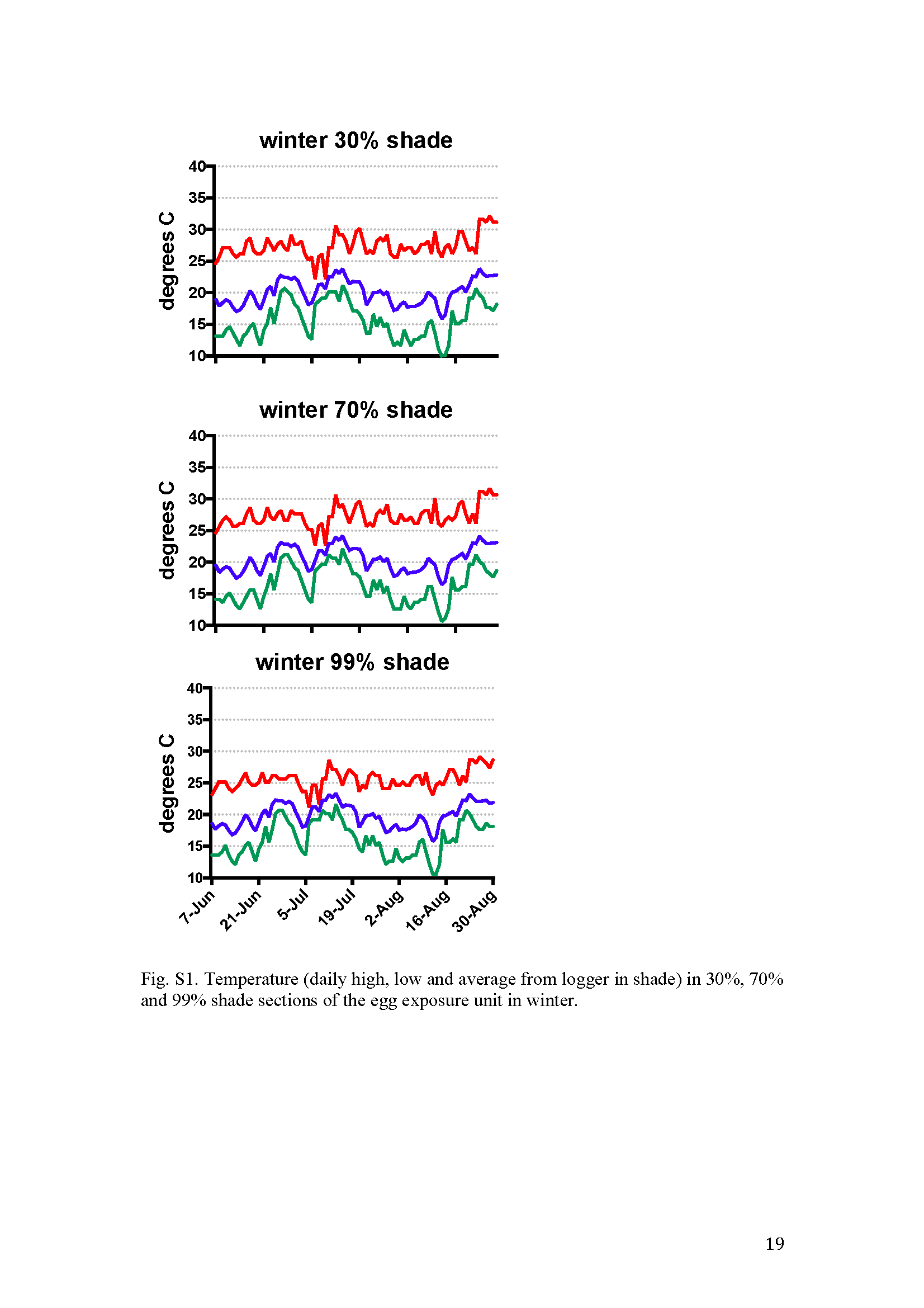

Supplement: S1 Fig — (TIFF) [file pntd.0003930.s001.tiff]

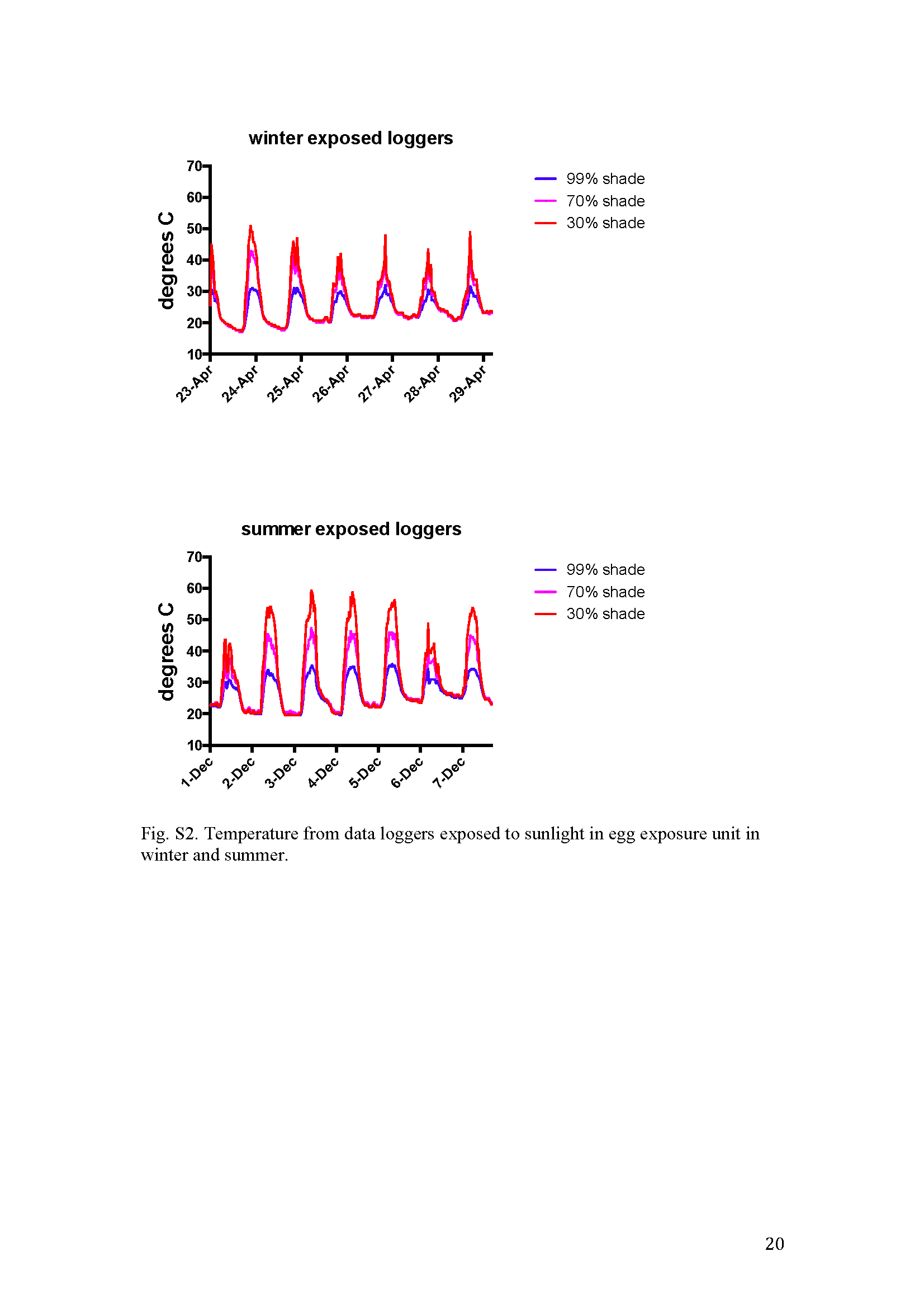

Supplement: S2 Fig — (TIFF) [file pntd.0003930.s002.tiff]

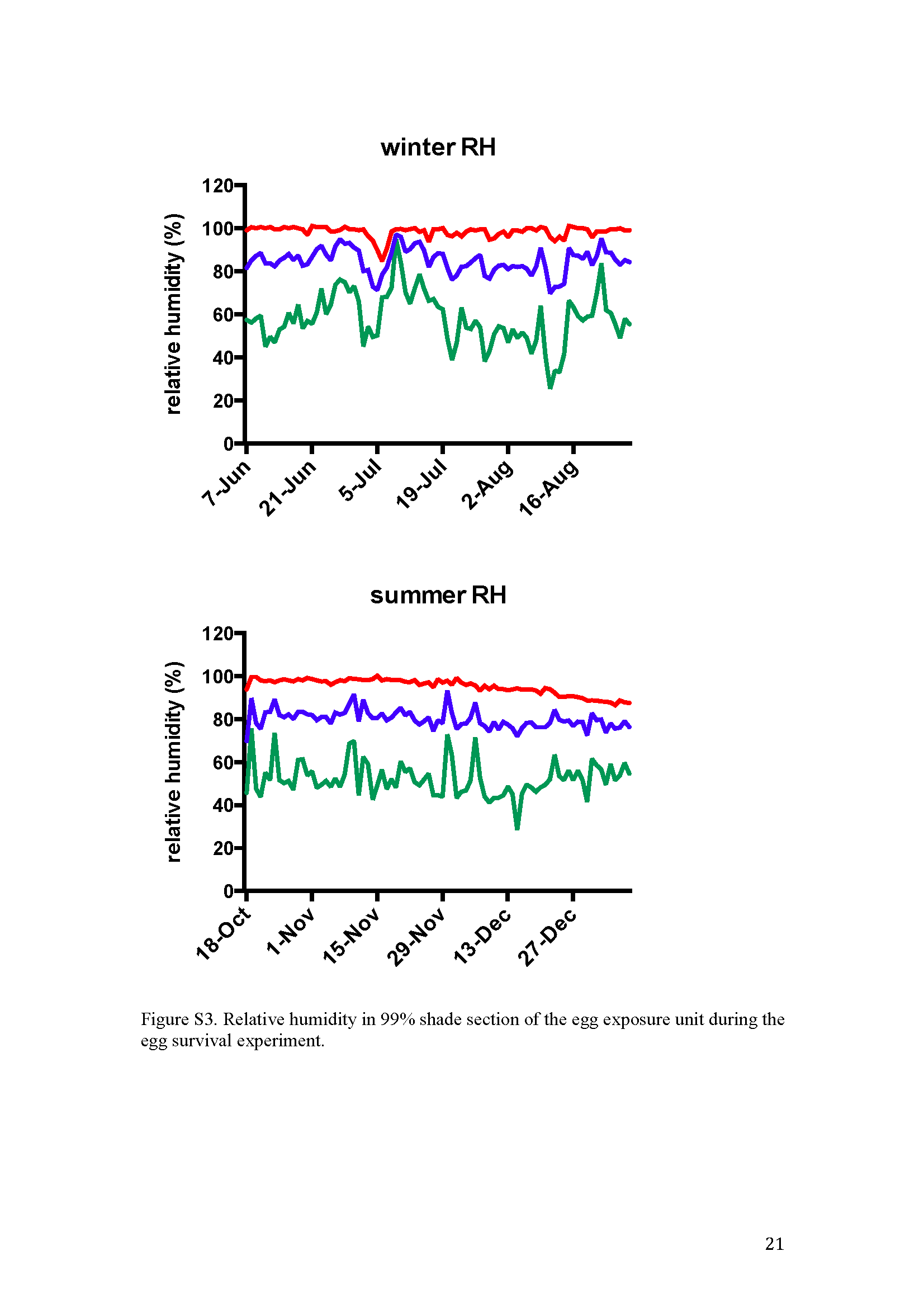

Supplement: S3 Fig — (TIFF) [file pntd.0003930.s003.tiff]

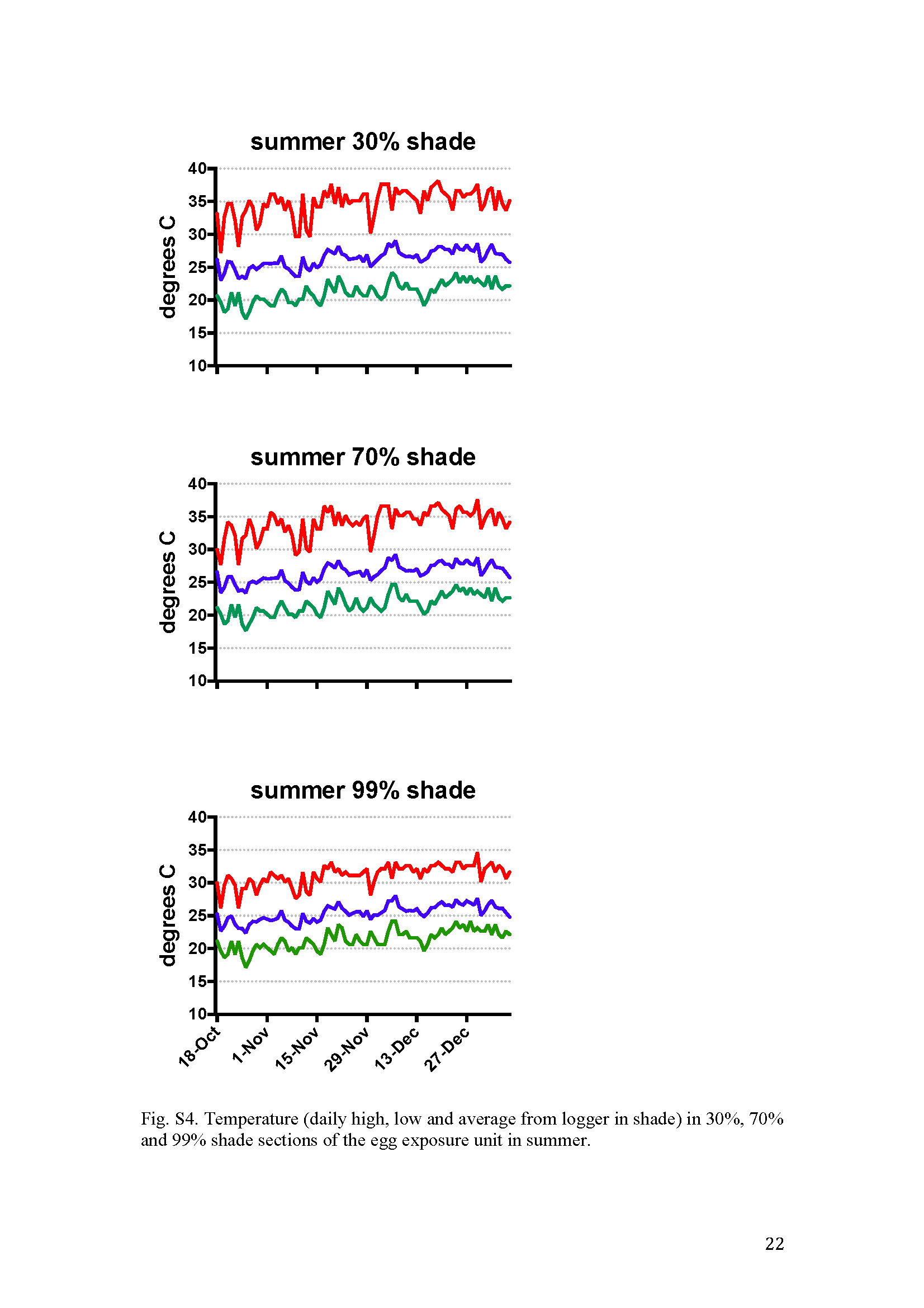

Supplement: S4 Fig — (TIFF) [file pntd.0003930.s004.tiff]

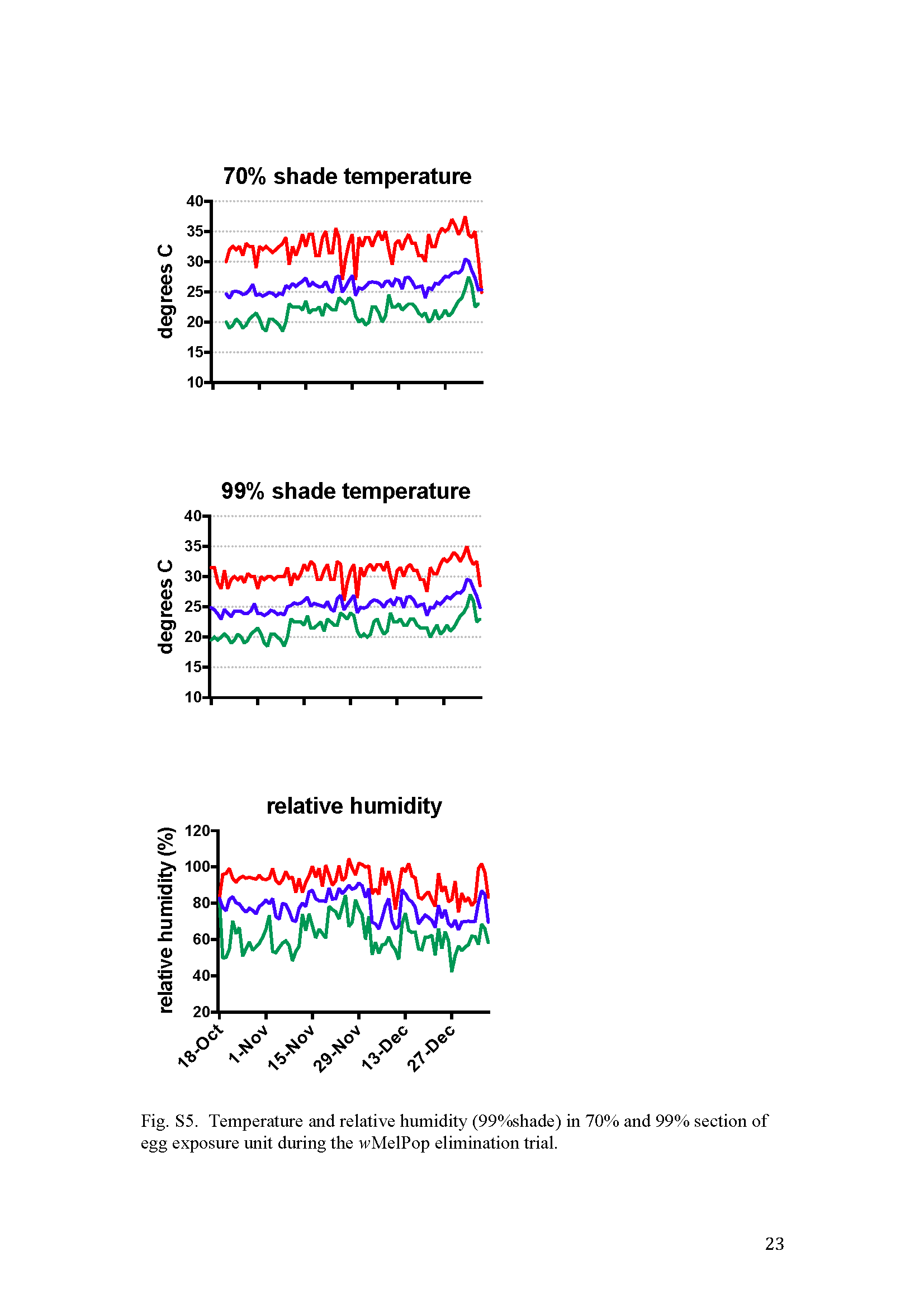

Supplement: S5 Fig — (TIFF) [file pntd.0003930.s005.tiff]
